# First-line penpulimab combined with anlotinib achieves complete response in an elderly patient with occult lung squamous cell carcinoma: a case report

**DOI:** 10.3389/fonc.2026.1841256

**Published:** 2026-07-14

**Authors:** Xiaoling Zhu, Yinan Yu, Wei Si, Jun Xu

**Affiliations:** 1 Department of Oncology, Shaoxing People's Hospital, The First Hospital of Shaoxing University, Shaoxing, Zhejiang, China; 2 Department of Radiation Oncology, Shaoxing People's Hospital, The First Hospital of Shaoxing University, Shaoxing, Zhejiang, China

**Keywords:** anlotinib, complete response, lung squamous cell carcinoma, occult, penpulimab

## Abstract

Occult lung cancer is a rare subtype of non-small cell lung cancer (NSCLC), characterized as an aggressive tumor with a poor prognosis. Its diagnosis relies on biopsy and pathological examinations. A durable complete response (CR) in advanced NSCLC achieved with a chemo-free and radiotherapy-free regimen remains exceedingly rare, particularly in elderly patients with a high tumor burden and unknown PD-L1 status. Here, we report an elderly patient who achieved CR of occult lung squamous cell carcinoma (LUSC) after treatment with a combination of penpulimab and anlotinib as first-line therapy. An 83-year-old man with occult LUSC (cTxN3M0, stage IIIB) presented with a mediastinal mass and multiple mediastinal and bilateral supraclavicular lymph node metastases, confirmed by supraclavicular lymph node examination biopsy. The patient was treated with a combination of penpulimab and anlotinib under close safety monitoring, resulting in a rapid tumor response and complete remission after ten cycles. This remission lasted over two years with no adverse effects observed during or after treatment. The therapy with penpulimab and anlotinib was continued for 26 months before being stopped at the patient’s request. These results suggest that penpulimab plus anlotinib may be a feasible first-line treatment option for elderly patients with advanced LUSC who cannot tolerate or choose to decline standard therapies. Notably, PD-L1 expression was not assessed because the patient refused testing, which prevents definitive conclusions regarding predictive factors associated with the observed response.

## 1 Introduction

Occult lung cancer, a rare subtype representing about 2% of non-small cell lung cancer (NSCLC), presents considerable diagnostic difficulties. Due to the absence of detectable pulmonary masses on imaging, diagnosis depends on biopsy and pathology. This form of lung cancer often displays aggressive characteristics, including rapid growth, early spread, and poor prognosis (1).

Immune checkpoint inhibitors (ICIs) have recently become a groundbreaking treatment approach for NSCLC (2). Immunotherapy targeting the programmed cell death protein 1 (PD-1)/programmed death ligand 1 (PD-L1) pathway has become an increasingly common first-line treatment option, used either alone or combined with chemotherapy (3, 4). Penpulimab, a distinctive IgG1 monoclonal antibody targeting PD-1, has gained exclusive approval in China for lung squamous cell carcinoma (LUSC) (5). It features a genetically engineered Fc segment and an optimized Fab segment, which could greatly decrease immune-related adverse effects (6–8). Anlotinib, a potent oral inhibitor of multiple tyrosine kinase targets, blocks angiogenesis by inhibiting vascular endothelial growth factor receptor 2 (VEGFR2), platelet-derived growth factor receptor-beta (PDGFR-β), and fibroblast growth factor receptor 1 (FGFR1) signaling pathways (9). The ALTER-0303 study confirmed that anlotinib could serve as a standard third-line therapy for patients with NSCLC (10).

Recently, the combination of immunotherapy and anti-angiogenic targeted treatments has shown promising results in clinical settings (11–14). Growing evidence confirms the clinical effectiveness of combined penpulimab and anlotinib therapy in malignancies (15, 16). This case illustrates the antitumor effect of CR using penpulimab combined with anlotinib as first-line therapy in an elderly patient diagnosed with mediastinal-type occult LUSC.

## 2 Patient information

An 83-year-old man arrived at our hospital in late August 2023, experiencing hoarseness and a mild cough for 10 days. His medical background includes hypertension and atrial fibrillation. He was on daily treatment with valsartan/hydrochlorothiazide 80/12.5 mg for hypertension and rivaroxaban 10 mg daily for anticoagulation, maintaining a heart rate around 80 bpm. He had a 30-pack-year smoking history, with no reported family history of relevant malignancy.

## 3 Diagnostic assessment and therapeutic intervention

**Figure 1:**
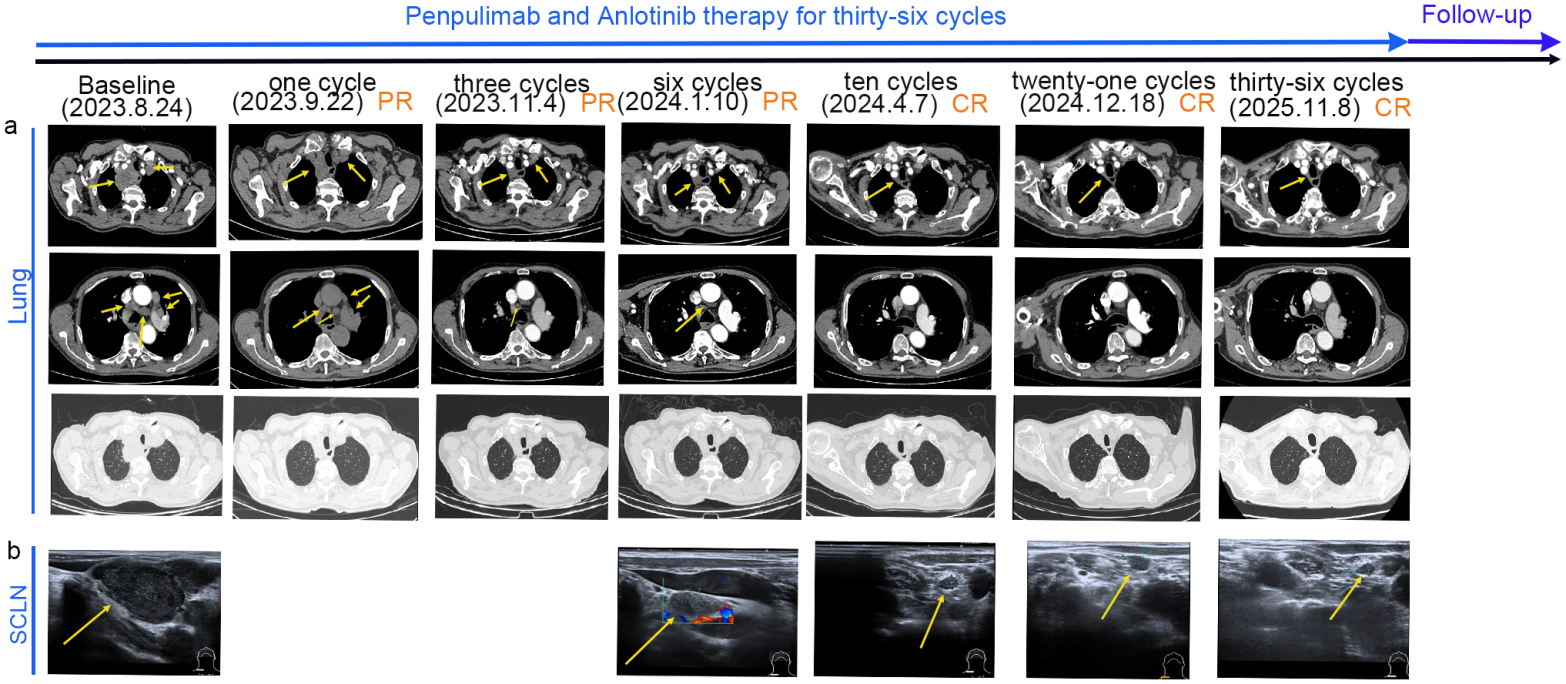
Representative images for each period, including pulmonary computed tomography (CT) **(a)** and ultrasonography of supraclavicular lymph nodes. **(b)** Positive staining for P63 (original magnification, ×100). Yellow arrows indicate the tumors. SCLN, supraclavicular lymph node; P, Penpulimab; A, Anlotinib; PR, partial response. CR, complete response.

**Figure 2:**
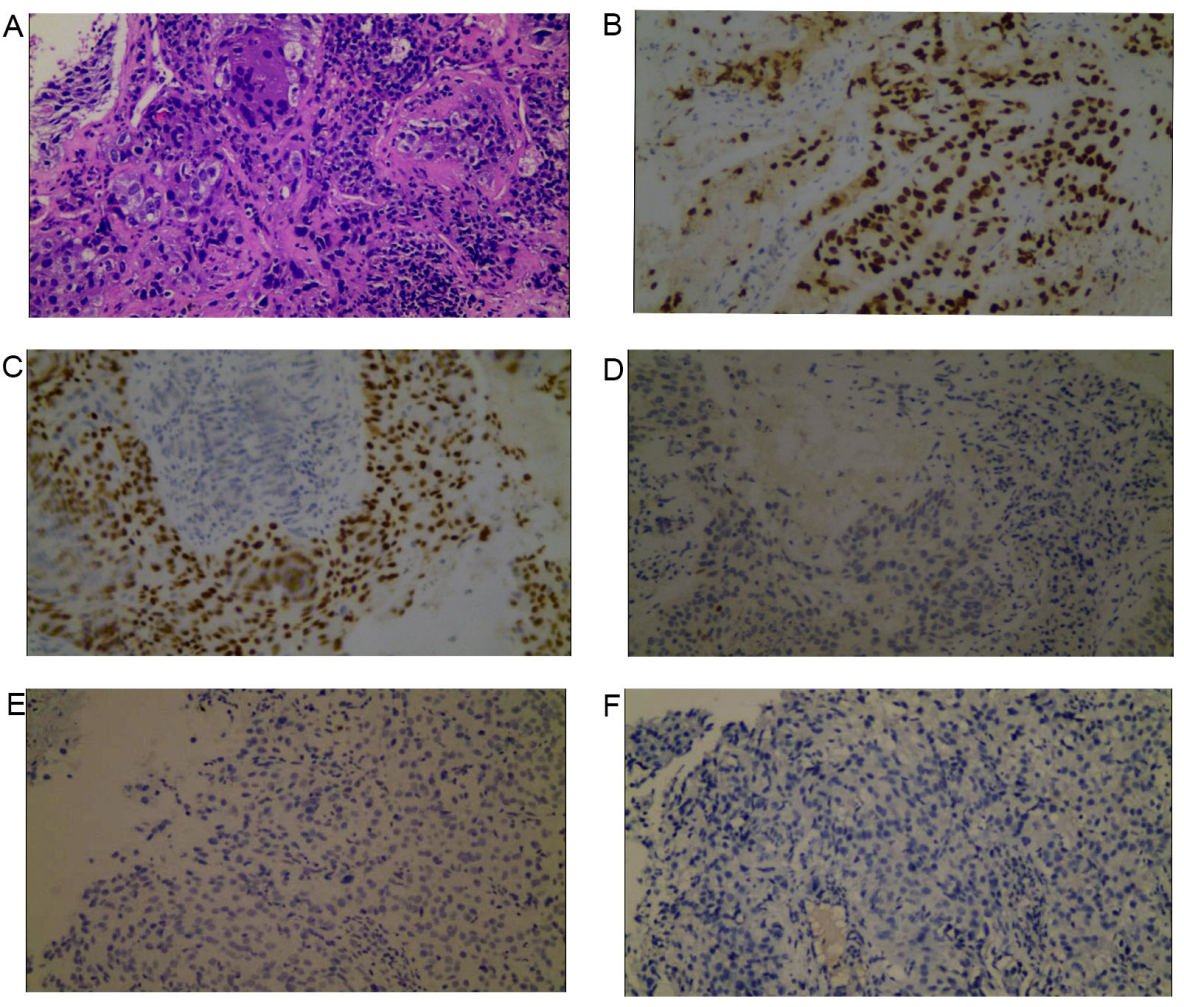
Immunohistochemical analysis of biopsy specimens from the right supraclavicular lymph node. **(A)** Hematoxylin and eosin (H&E) staining revealed nested proliferation with low differentiation and occasional intercellular bridges, confirming metastatic poorly differentiated squamous cell carcinoma (original magnification, ×100). **(B)** Positive staining for P63 (original magnification, ×100). **(C)** Positive staining for P40 (original magnification, ×100). **(D)** Negative staining for TTF-1 (original magnification, ×100). **(E)** Negative staining for CD5 (original magnification, ×100). **(F)** Negative staining for CD117 (original magnification, ×100). The morphology seen in H&E and the lack of Synaptophysin (Syn) staining exclude a neuroendocrine tumor. P63 and P40 positivity support squamous differentiation, TTF-1 negativity argues against pulmonary adenocarcinoma, and negative staining for CD5 and CD117 helps rule out thymic origin.

**Figure 3:**
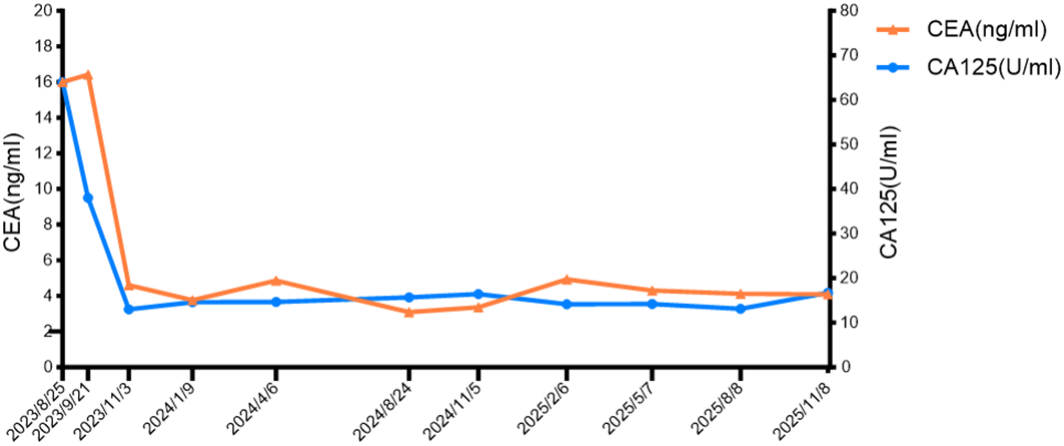
Changes in serum CEA and CA125 levels during treatment. CEA, carcinoembryonic antigen; CA125, cancer antigen 125.

On August 24, 2023, enhanced chest computed tomography (CT) showed multiple mediastinal lymph nodes fused together, forming an irregular 46×37 mm mass in the right mid-upper mediastinum. This mass displayed heterogeneous density and enhancement, with no pulmonary space-occupying lesion identified (**Figure 1A**, 1st column). Laryngoscopy performed at presentation revealed right vocal cord paralysis without any mucosal lesion, mass, or intrinsic laryngeal or hypopharyngeal tumor. Brain MRI and contrast-enhanced chest CT showed no nasopharyngeal mass, esophageal wall thickening, or abnormal enhancement. Physical examination also revealed no lesions in the oropharynx or oral cavity. Ultrasound revealed multiple bilateral supraclavicular lymph nodes, with the largest measuring 29×19 mm on the right and 20×13 mm on the left, both showing loss of corticomedullary differentiation, suggesting metastasis (**Figure 1B**, 1st column). Follow-up brain MRI, bone scintigraphy, and abdominal CT scans revealed no evidence of distant metastases. Due to financial constraints, the patient and his family declined further PET-CT examination. A biopsy of the right supraclavicular lymph node confirmed metastatic poorly differentiated squamous cell carcinoma. In the absence of any identifiable primary lesion in the lung parenchyma, upper aerodigestive tract, or other sites, the immunohistochemical profile supported pulmonary origin as the most probable diagnosis. Microscopic examination showed tumor cells arranged in nested patterns with low differentiation and some intercellular bridges. Immunohistochemical results were positive for p63 and p40, and negative for TTF-1, Syn, CgA, CD5, and CD117. The Ki-67 proliferation index was 60%. (**Figure 2**). Serum levels of carcinoembryonic antigen (CEA) and cancer antigen 125 (CA-125) were measured at 16.01 ng/ml (reference <5) and 64.03 U/ml (reference <35), respectively (see **Figure 3**). The patient’s performance status was assessed as grade 1 based on the Eastern Cooperative Oncology Group (ECOG) criteria. Physical examination identified enlarged bilateral supraclavicular lymph nodes, with the largest measuring 3×3 cm; they were firm, fixed, and non-tender. Cardiopulmonary and abdominal examinations showed no abnormalities.

Considering the patient’s limited tolerance for invasive procedures and his refusal of further diagnostic interventions, bronchoscopy was not performed, particularly because contrast-enhanced chest CT showed no endobronchial lesions. Although PD-L1 expression testing on the biopsy specimen was technically feasible and is recommended as a category 1 indication in the NCCN guidelines, the patient explicitly declined the test because of financial concerns. He also declined molecular testing for actionable driver alterations, as such alterations are rarely detected in patients with squamous histology and a heavy smoking history. Given his advanced age and comorbidities, he further refused thoracoscopic biopsy and conventional chemoradiotherapy. Based on the pathological findings and multidisciplinary team discussion, he was diagnosed with occult LUSC with mediastinal presentation (cTxN3M0, stage IIIB). Considering his advanced age, comorbidities, and refusal of conventional chemoradiotherapy, first-line treatment with penpulimab combined with anlotinib was initiated on August 30, 2023, after thorough communication and informed consent from the patient and his family. The treatment regimen consisted of penpulimab 200 mg administered intravenously every three weeks and anlotinib 8 mg taken orally once daily for two weeks in each three-week cycle. Notably, anlotinib was started at a reduced dose of 8 mg rather than the standard 12 mg used in ALTER-0303 to reduce the risk of adverse events while maintaining the expected antitumor effect. Given the patient’s advanced age and comorbidities, cardiovascular safety was monitored closely throughout treatment. Blood pressure was checked weekly, and electrocardiographic and coagulation assessments were performed at baseline and before each treatment cycle.

## 4 Follow-up and outcomes

Remarkably, after one treatment cycle, all lesions significantly decreased in size, with the mediastinal mass shrinking to 32×22 mm, achieving a Partial Response (PR) (**Figure 1A**, 2nd column), with CA125 showing a notable decrease, while CEA levels exhibited a minimal rise (**Figure 3**). After three cycles, the tumor further regressed to 23×18 mm (**Figure 1A**, 3rd column), with tumor markers normalized (CEA 4.6 ng/ml, CA-125 13.02 U/ml) (**Figure 3**). During treatment, continued shrinkage of bilateral vascular nodes was observed through physical examination and ultrasound. Hoarseness resolved, and the voice returned to normal after six cycles of treatment, with the performance status improving from ECOG 1 to ECOG 0. Ten cycles later, no residual tumors were observed in the mediastinum (**Figure 1A**, fifth column) or in the supraclavicular lymph nodes (right 6×4 mm, left 5×4 mm) (**Figure 1B**, fifth column). This results in a complete response (CR) based on the Response Evaluation Criteria in Solid Tumors, version 1.1 (RECIST 1.1).

The patient completed 36 cycles of treatment over 26 months (final dose: November 8, 2025) with excellent tolerability, and no immune-related or antiangiogenic-related adverse events were observed. To date, the patient has maintained consistently normal tumor marker levels (**Figure 3**), and there is no imaging evidence of recurrence on regular CT scans during and after treatment (**Figure 1**).

## 5 Discussion

Globally, occult lung cancer is a complex and difficult clinical problem, accounting for about 2% of NSCLC cases. It usually progresses rapidly, spreads early, and has a poor prognosis (1). When no pulmonary space-occupying lesion is present, diagnosis depends largely on accurate tissue biopsy and pathological examination. In this case, the patient exhibited hoarseness and a mild cough. Enhanced CT scans showed a mass in the right upper to middle mediastinum, along with multiple mediastinal lymph node metastases, but no lung lesions were detected. Ultrasound confirmed metastases in bilateral supraclavicular lymph nodes. The mediastinal mass required differentiation from other mediastinal tumors, including thymic carcinoma, pleural mesothelioma, and lymphoma, as well as primary squamous cell carcinoma arising from the upper aerodigestive tract (UADT). Immunohistochemical analysis of the right supraclavicular lymph node biopsy showed P63(+), p40(+), TTF-1(−), Syn(−), CD5(−), and CD117(−), with a Ki-67 proliferation index of 60%. These findings were consistent with metastatic poorly differentiated squamous cell carcinoma of most likely pulmonary origin. Positivity for P63 and p40 supported squamous differentiation. TTF-1 negativity excluded pulmonary adenocarcinoma. H&E morphology together with Syn negativity ruled out a neuroendocrine tumor, while CD5 and CD117 negativity rendered thymic carcinoma unlikely, though not definitively excluded. Subsequent TdT and PAX8 staining were both negative (**Supplementary Material**). Importantly, a systematic evaluation was carried out to rule out extrapulmonary primary tumors. Physical examination showed no primary lesion in the oropharynx. Laryngoscopy excluded an intrinsic laryngeal or hypopharyngeal tumor. Contrast-enhanced CT revealed no esophageal or other UADT lesion, and brain MRI showed no nasopharyngeal mass. Panendoscopy was not performed because of the patient’s advanced age and refusal of further invasive procedures. However, the comprehensive evaluation did not identify a UADT primary site, which was consistent with occult lung cancer (cTxN3M0). In addition, the patient presented with hoarseness, and laryngoscopy demonstrated paralysis of the right vocal cord. Following treatment, the mediastinal mass decreased in size, and the patient’s voice completely recovered after six treatment cycles.

This finding suggests that the hoarseness was most likely caused by compression of the right recurrent laryngeal nerve by the large fused mediastinal lymph node mass at the mediastinal level. The nerve loops around the right subclavian artery within the superior mediastinum. The complete resolution of hoarseness after six cycles of treatment, together with marked shrinkage of the mediastinal mass, further supports this explanation.

Over the past decade, the advent of immunotherapy has changed the treatment landscape for inoperable NSCLC without driver mutations by blocking the PD-1/PD-L1 pathway, reversing T-cell suppression, and remodeling antitumor immunity responses (17, 18). Based on clinical trials such as KEYNOTE-042 (19) and KEYNOTE-407 (20), immunotherapy, either alone or combined with chemotherapy, is now considered the standard first-line treatment (3). PD-L1 expression is the only biomarker approved by the FDA for guiding immunotherapy (21). Several studies have shown that in patients with high PD-L1 expression (≥50%), immunotherapy considerably extends progression-free survival (PFS) and overall survival (OS) compared to chemotherapy, while also causing fewer adverse effects (2, 22). However, reports of CR with ICIs alone or in combination for advanced NSCLC are still uncommon. In the KEYNOTE-024 trial, only 6 of 154 patients (3.8%) with PD-L1 ≥50% achieved CR with pembrolizumab (2). A meta-analysis of clinical trials found a low CR rate of just 1.5% with ICIs alone or combined with chemotherapy. This suggests that eliciting deep responses remains a significant clinical challenge, even among patients with PD-L1≥50% (23). Notably, durable CR achieved with a chemo-free and radiotherapy-free regimen is even less common. In published studies evaluating anti-PD-1 therapy combined with anti-angiogenic agents without chemotherapy for advanced NSCLC, such as sintilimab plus anlotinib (13), CR rates have rarely been reported and are estimated to be below 5%. Sustained CR lasting longer than 12 months is considered an exceptional outcome. To our knowledge, this is the first reported case of a chemo-free and radiotherapy-free penpulimab-anlotinib combination achieving durable CR lasting more than 26 months in an elderly patient with occult LUSC and a high mediastinal tumor burden.

Angiogenesis is a key factor in tumor development and metastasis (24). The irregular structure and function of tumor blood vessels limit blood flow and perfusion, leading to hypoxia and acidosis, which inhibit dendritic cell (DC) maturation and increase PD-L1 expression, ultimately establishing an immunosuppressive tumor microenvironment (TME) (11). Anti-angiogenesis therapy not only inhibits the blood vessels necessary for tumor growth and spread but also alters the tumor immune microenvironment (11, 25, 26). Moreover, by stimulating T cells, ICIs also normalize tumor vessels, which in turn promotes effector T cell infiltration, leading to a more favorable TME remodeling (12, 25, 27). Thus, combining anti-angiogenic therapy with immunotherapy produces synergistic effects and enhances treatment outcomes (12). Anlotinib, a potent multi-targeted agent, inhibits tumor growth by blocking angiogenesis, cell proliferation, and metastasis (9). The ALTER-0303 clinical trial demonstrated that anlotinib significantly improved median PFS by 4 months (hazard ratio [HR], 0.25), with median OS increasing from 6.3 to 9.6 months (HR, 0.68), compared with placebo in third-line treatment (10). Multiple clinical trials have shown that Anlotinib, combined with anti-PD-1 therapy, has promising antitumor effects as both a first-line and later treatment option (13, 14, 28).

Penpulimab, an IgG1 monoclonal antibody targeting PD-1, has gained exclusive approval in China for LUSC (5). Compared with IgG4 subtypes, IgG1 antibodies are more stable, reducing the risk of aggregation and preventing tumor immune escape. Additionally, by modifying the Fc segment and optimizing the Fab segment, the Fc effect can be silenced, preventing the phagocytosis or killing of immune T cells and decreasing IL-8 release (6–8). Therefore, penpulimab not only boosts the effectiveness of immunotherapy but also greatly enhances drug safety (7). The combination of penpulimab and anlotinib has been extensively studied in certain cancers, showing promising results (15, 16). Laboratory studies have shown that LUSC exhibits greater immune heterogeneity than adenocarcinoma, with more abundant Treg cells and irregular activation of inflammation-associated signaling pathways (29). The decision to initiate first-line penpulimab plus anlotinib was made after MDT discussion and was based on several patient-specific and evidence-based considerations. First, the patient declined concurrent chemoradiotherapy because of his advanced age and comorbidities. This treatment remains the guideline-recommended standard for unresectable stage IIIB NSCLC according to the PACIFIC trial. He also refused PD-L1 testing and invasive bronchoscopy, which prevented PD-L1-guided treatment selection. Second, the unknown PD-L1 status and high mediastinal tumor burden suggested that the response to ICI monotherapy might be limited. Therefore, anlotinib was added because of its established ability to remodel the immunosuppressive TME through anti-angiogenic mechanisms, which may enhance the efficacy of immunotherapy in this setting (11, 25, 26). Third, penpulimab was selected because its engineered Fc segment provides a favorable safety profile that may be particularly suitable for elderly patients (7, 8). Fourth, phase Ib data from sintilimab plus anlotinib used as a chemo-free first-line treatment for advanced NSCLC (13) provided clinical support for the anti-PD-1 plus anlotinib treatment strategy. Remarkably, the tumor regressed rapidly after only one treatment cycle. Of note, CEA levels increased transiently and slightly after the first cycle despite radiological evidence of partial response and returned to normal by the third cycle. This pattern may reflect early treatment-induced tumor cell death rather than disease progression. The patient achieved CR after ten cycles of treatment, approximately seven months after therapy initiation, and has maintained PFS for 26 months to date. No adverse events related to immunotherapy or targeted therapy were observed throughout the treatment period. This favorable outcome may be associated with the optimized Fab segment and genetically engineered Fc segment of penpulimab (7, 8), which can significantly reduce immune-related adverse events. Meanwhile, anlotinib was administered at a reduced dose of 8 mg daily instead of the standard 12 mg because of the patient’s advanced age, comorbidities, and concurrent use of rivaroxaban for anticoagulation. Real-world evidence suggests that reduced-dose anlotinib (8-10 mg) can maintain antitumor activity while offering better tolerability in elderly patients. No targeted therapy-related adverse effects, including uncontrolled hypertension, proteinuria, or bleeding, were observed during treatment. Given the potential CYP3A4/P-gp interaction with rivaroxaban, coagulation parameters were monitored regularly. No bleeding events occurred, and the patient maintained a good quality of life throughout treatment.

In this study, the patient’s age of 83 years is particularly noteworthy. In the present report, “elderly” refers to patients aged ≥75 years, consistent with the criteria used in the cited studies (30–32). Traditional chemoradiotherapy often shows limited effectiveness and poor tolerability among elderly patients with multiple health issues, highlighting the need for more appropriate treatment options. Large-scale clinical trial data on patients over 75 or even 80 years old are limited. A real-world retrospective study involving 290 elderly patients with advanced NSCLC, including 16 patients over 80, demonstrated that advanced age does not reduce the treatment effectiveness of ICIs (30). The IPSOS study, the first and only prospective phase III trial worldwide to include advanced NSCLC patients who are intolerant to platinum-based chemotherapy, enrolled 453 patients with a median age of 75; 31% were over 80 years old. The results showed that atezolizumab monotherapy significantly improved overall survival compared with single-agent chemotherapy (median overall survival: 10.3 months versus 9.2 months), increased the 2-year survival rate from 12% to 24%, indicating a benefit independent of PD-L1 status, and improved patients’ quality of life (31). Meta-analyses also show that for elderly patients aged ≥75 years, ICIs provide a significant survival benefit in first-line treatment (HR 0.78), but have no significant effect in second-line treatment (HR 1.02), indicating that immunotherapy should be used as the first-line therapy in elderly patients (32). At the mechanistic level, increased lymphocyte infiltration and relatively low Treg levels in the tumor microenvironment of elderly patients may explain the effectiveness of immunotherapy (33, 34). Based on this evidence, first-line immunotherapy was initiated. Because PD-L1 expression data were unavailable, the mediastinal tumor burden was high, and LUSC is known to have a complex tumor immune microenvironment, anlotinib was added as targeted therapy (35). Careful safety monitoring was conducted throughout treatment, and a marked clinical benefit was achieved. Although definitive biomarker data were unavailable, the biological basis for the observed CR in this PD-L1-unknown setting may be partly explained by the TME-remodeling effects of anlotinib. By reducing PD-L1 expression on vascular endothelial cells and promoting effector T cell infiltration (26), anlotinib may enhance the efficacy of immunotherapy regardless of baseline PD-L1 status. This possibility is supported by phase Ib data showing that sintilimab plus anlotinib achieved an ORR of 72.7% irrespective of PD-L1 expression level (13). In addition, immune characteristics specific to LUSC, including higher immune cell infiltration and relatively lower Treg levels in elderly patients (33, 34), may also have contributed to the favorable response observed in this case.

This study provides the first reported evidence that a chemo-free and radiotherapy-free regimen consisting of penpulimab combined with anlotinib can achieve deep and durable CR lasting more than 26 months in an 83-year-old patient with occult LUSC and a high mediastinal tumor burden. Although CR achieved with immunotherapy-based treatment is generally considered notable, durable CR achieved without either chemotherapy or radiotherapy represents a distinctly uncommon clinical outcome. The current standard treatment for unresectable stage IIIB NSCLC remains concurrent chemoradiotherapy followed by durvalumab consolidation according to the PACIFIC regimen (36). However, this approach is often difficult for elderly patients with multiple comorbidities to tolerate. Our case suggests that a well-tolerated chemo-free combination may be capable of overcoming the immunosuppressive tumor microenvironment associated with unknown PD-L1 status and high tumor burden, leading to outcomes beyond those typically expected from chemotherapy-free approaches. This result may provide a potential treatment option for patients facing similar clinical challenges. However, several limitations should be acknowledged. First, the use of penpulimab plus anlotinib as first-line treatment represents a non-standard and off-label strategy for stage IIIB NSCLC. Its use was based on individualized clinical considerations and supported by emerging mechanistic and clinical evidence. However, it should not be interpreted as a general treatment recommendation without prospective validation. Second, key biomarker data were unavailable, including pretreatment PD-L1 expression, Tumor Mutation Burden (TMB), and Tumor Infiltrating Lymphocyte (TIL) density, which limited our ability to evaluate predictive factors associated with treatment efficacy. Third, because of patient and family preferences, concerns regarding treatment tolerance, and financial limitations, neither PET-CT nor bronchoscopy was performed. These limitations may have affected the completeness of staging and assessment of the primary tumor site. In addition, anlotinib was administered at a reduced dose of 8 mg daily rather than the standard 12 mg dose used in ALTER-0303. This limits direct comparison with existing clinical trial data and prevents firm conclusions regarding the contribution of dose intensity to the observed complete response. Although the outcome in this individual case is encouraging, caution is needed when applying these findings more broadly. High-quality clinical studies are still required to confirm the safety and potential benefits of combining penpulimab and anlotinib as first-line treatment for elderly patients with advanced LUSC.

In conclusion, we report an 83-year-old patient who achieved rapid tumor regression and durable CR lasting more than 26 months for occult locally advanced LUSC with a high mediastinal tumor burden following first-line treatment with penpulimab plus anlotinib, without chemotherapy or radiotherapy. This outcome is particularly noteworthy given the patient’s advanced age, unknown PD-L1 status, and multiple comorbidities. Although the generalizability of a single case is inherently limited, this combination may represent a feasible treatment option for elderly patients with advanced NSCLC who cannot tolerate or choose to refuse chemoradiotherapy. Prospective studies involving larger patient populations are needed to optimize dosing strategies, confirm efficacy, and identify predictive biomarkers for patient selection.

Ethics Statement and Informed Consent: This case report was conducted in accordance with the Declaration of Helsinki. Ethical approval for publication of this case was obtained from the Academic Ethics Committee of Shaoxing People’s Hospital (Approval No. [2026-008]). Written informed consent was obtained from the patient and his legal family representative for the off-label use of penpulimab plus anlotinib and for the publication of the clinical data, images, and follow-up information included in this report.

## Data Availability

The original contributions presented in the study are included in the article/**Supplementary Material**. Further inquiries can be directed to the corresponding author.
