# Supplementary figures and images for: First-line penpulimab combined with anlotinib achieves complete response in an elderly patient with occult lung squamous cell carcinoma: a case report
Source: Front Oncol. 2026 Jul 14;16:1841256. doi: 10.3389/fonc.2026.1841256 (PMC13407116; doi:10.3389/fonc.2026.1841256)

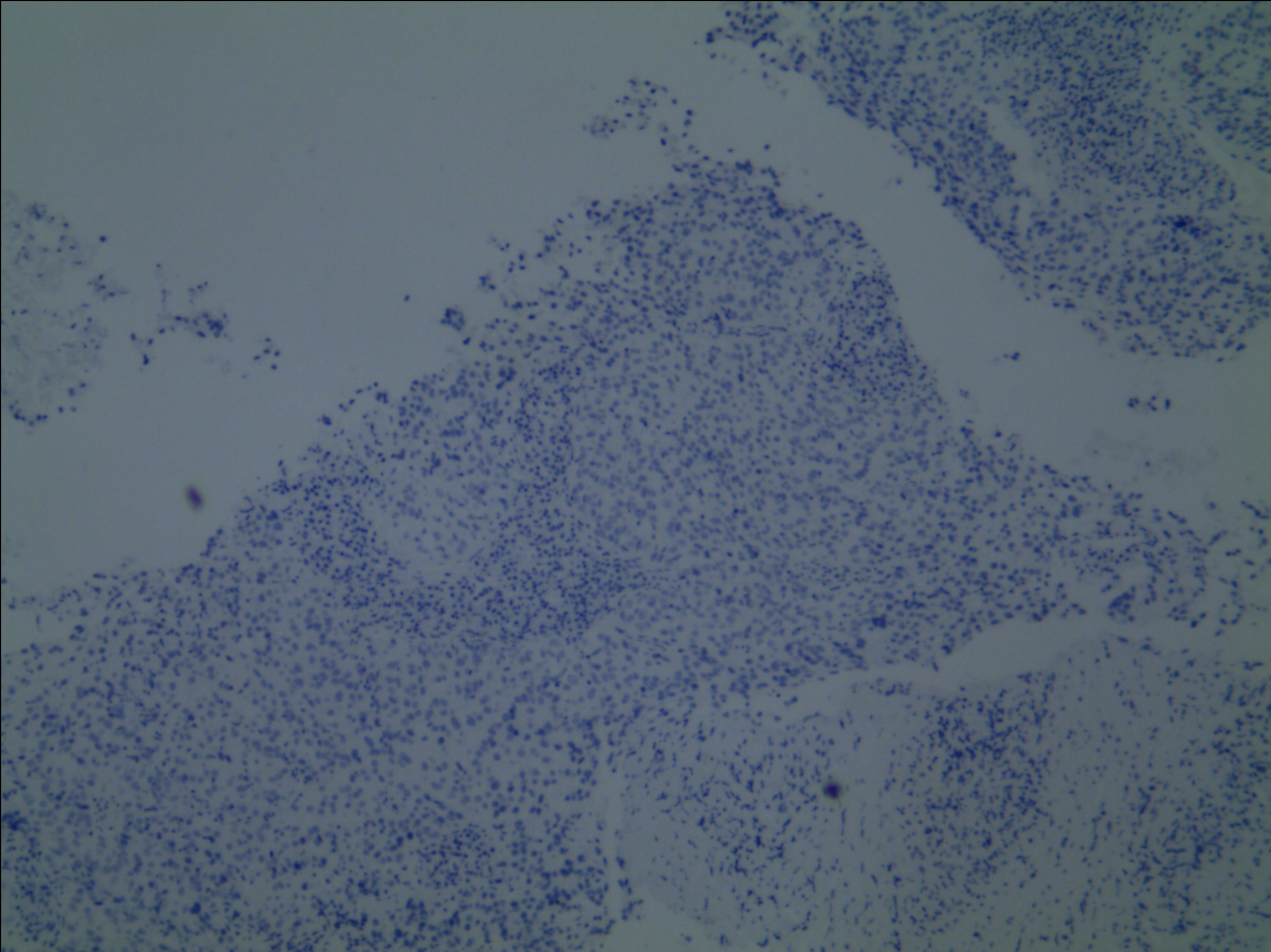

Supplement: Supplementary file 1 [file Image1.png]

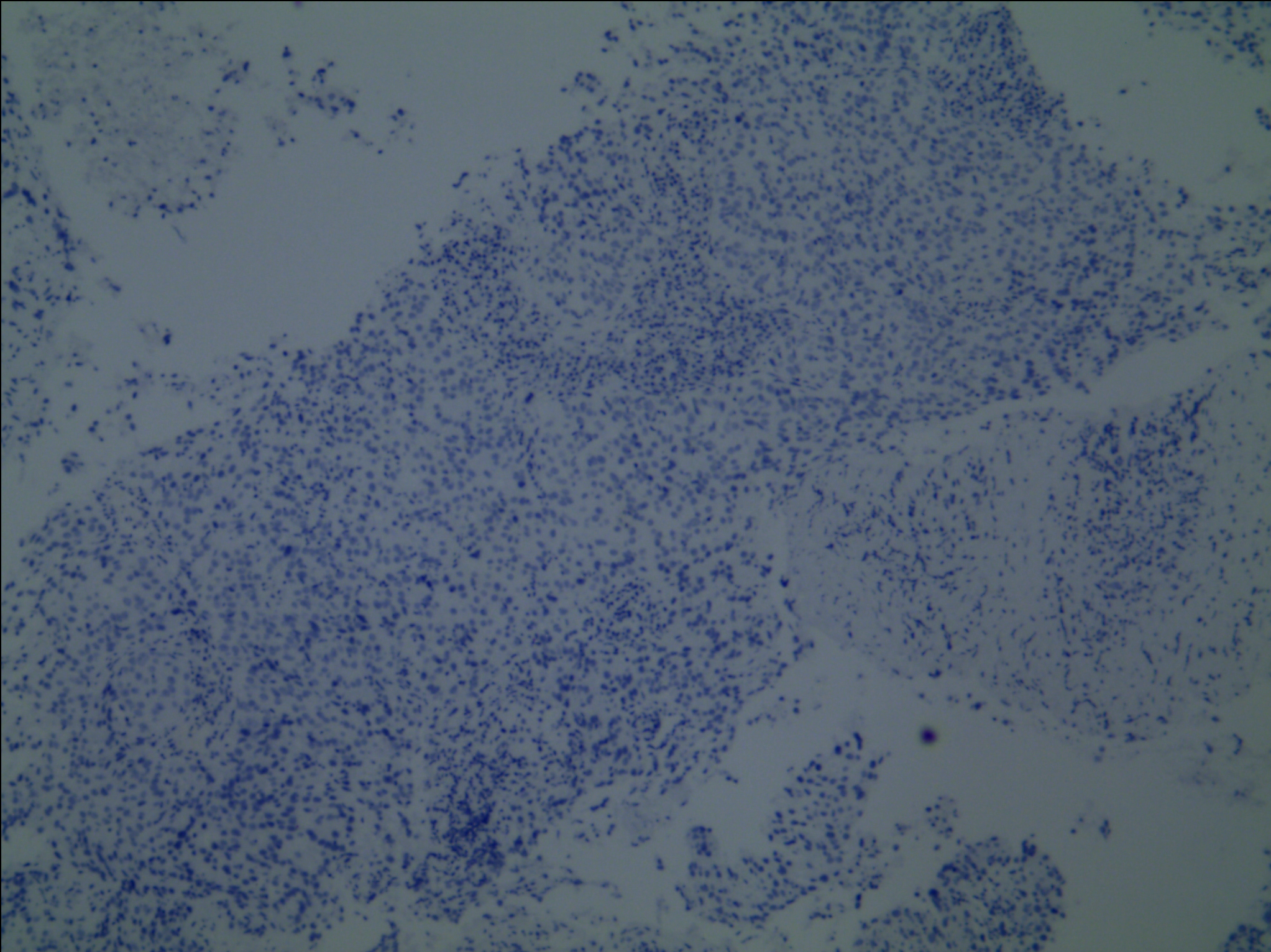

Supplement: Supplementary file 2 [file Image2.png]
